# Daratumumab‐lenalidomide‐dexamethasone vs standard‐of‐care regimens: Efficacy in transplant‐ineligible untreated myeloma

**DOI:** 10.1002/ajh.25963

**Published:** 2020-09-05

**Authors:** Brian G. M. Durie, Shaji K. Kumar, Saad Z. Usmani, Bareng A. S. Nonyane, Eric M. Ammann, Annette Lam, Rachel Kobos, Eric M. Maiese, Thierry Facon

**Affiliations:** ^1^ Cedars‐Sinai Medical Center Los Angeles California USA; ^2^ Mayo Clinic Rochester Minnesota USA; ^3^ Atrium Health Charlotte North Carolina USA; ^4^ Johns Hopkins Bloomberg School of Public Health Baltimore Maryland USA; ^5^ Janssen Scientific Affairs Horsham Pennsylvania USA; ^6^ Janssen Global Services Raritan New Jersey USA; ^7^ Janssen Research & Development Raritan New Jersey USA; ^8^ Department of Haematology Lille University Hospital Lille France

## Abstract

Daratumumab in combination with lenalidomide‐dexamethasone (D‐Rd) recently received FDA approval for the treatment of transplant‐ineligible patients with newly diagnosed multiple myeloma (NDMM). The present PEGASUS study compared progression‐free survival (PFS) in patients treated with D‐Rd in the MAIA trial and patients treated with common standard‐of‐care regimens from the Flatiron Health electronic health record‐derived deidentified database, which has data from patients treated primarily at community‐based oncology practices in the United States. Individual‐level patient data from both data sources were used to perform an anchored indirect treatment comparison (ITC) of D‐Rd to bortezomib‐lenalidomide‐dexamethasone (VRd) and bortezomib‐dexamethasone (Vd); lenalidomide‐dexamethasone (Rd) was the common anchor for the ITC. Hazard ratios (HRs) reflecting direct comparisons of PFS within MAIA (D‐Rd vs Rd) and Flatiron Health (VRd vs Rd; Vd vs Rd) were used to make ITCs for D‐Rd vs VRd and Vd, respectively. After application of MAIA inclusion/exclusion criteria and propensity‐score weighting, the Flatiron Health patients resembled the MAIA trial population on measured baseline characteristics. Based on the direct comparison within MAIA, treatment with D‐Rd was associated with a significantly lower risk of progression or death compared to Rd (HR 0.54; 95% CI 0.42, 0.71). Based on the ITCs, D‐Rd was associated with a significantly lower risk of progression or death compared to VRd (HR 0.68; 95% CI 0.48, 0.98) and Vd (HR 0.48; 95% CI 0.33, 0.69). In the absence of head‐to‐head trials comparing D‐Rd to VRd or Vd, the present ITC may help inform treatment selection in transplant‐ineligible patients with NDMM.

## 1 INTRODUCTION

Daratumumab in combination with lenalidomide‐dexamethasone (D‐Rd) received approval from the US Food and Drug Administration (FDA) in June 2019 for the treatment of patients with newly diagnosed multiple myeloma (NDMM) who were ineligible for autologous stem cell transplant (ASCT).^1^ FDA approval was based on results from a prespecified interim analysis of data from the MAIA phase III clinical trial, which showed that D‐Rd was associated with a significantly lower risk of disease progression or death relative to lenalidomide‐dexamethasone (Rd) alone in patients with transplant‐ineligible NDMM (hazard ratio [HR] 0.56; 95% confidence interval [CI] 0.43, 0.73; *P* < .001).^1^, ^2^

In the US, common standard‐of‐care (SOC) regimens for the treatment of transplant‐ineligible NDMM include lenalidomide‐dexamethasone (Rd), bortezomib‐lenalidomide‐dexamethasone (VRd), and bortezomib‐dexamethasone (Vd), which together account for at least two‐thirds of treatment regimens used to treat patients with transplant‐ineligible NDMM.^3^, ^4^ In recent years, VRd has become a preferred regimen in patients with sufficient fitness for triplet therapy based on results from the SWOG S0777 trial, which demonstrated superior outcomes associated with VRd relative to Rd in NDMM patients without intent for immediate transplant.^5^ To date, no clinical trials have directly compared D‐Rd to SOC regimens other than Rd in patients with NDMM ineligible for transplant.^2^ To address this evidence gap, the present PEGASUS study compared progression‐free survival (PFS) in transplant‐ineligible NDMM patients treated with D‐Rd in the MAIA trial and similar patients treated with VRd or Vd in routine clinical practice settings.

## 2 METHODS

### 2.1 Study population and design

**FIGURE 1:**
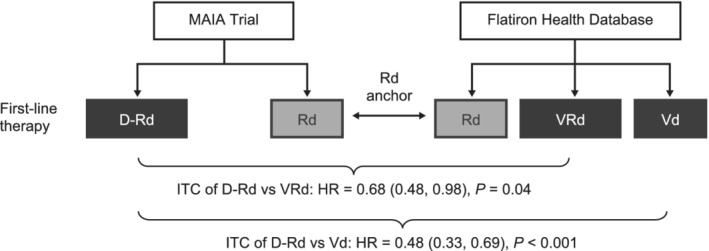
PEGASUS study design and progression‐free survival hazard ratios with 95% confidence intervals for D‐Rd relative to alternative standard‐of‐care regimens based on indirect treatment comparisons. D‐Rd, daratumumab‐lenalidomide‐dexamethasone; HR, hazard ratio; ITC, indirect treatment comparison; Rd, lenalidomide‐dexamethasone; Vd, bortezomib‐dexamethasone; VRd, bortezomib‐lenalidomide‐dexamethasone. Figure reflects results for primary on‐treatment analysis of progression‐free survival (PFS) with weighting of Flatiron Health treatment groups to resemble MAIA trial population

The present study (PEGASUS) used individual‐level patient data from the global MAIA phase III randomized controlled trial and the US Flatiron Health electronic health record (EHR)‐derived deidentified database (Figure S1 in Appendix S1). An anchored indirect treatment comparison (ITC) study design was used to compare relative treatment effects across the two data sources (Figure 1).^6^, ^7^ Details on the data sources and methodology are provided below.

#### 2.1.1 MAIA trial

Patients in MAIA were enrolled between March 2015 and January 2017 and randomized to receive D‐Rd or Rd. Key eligibility criteria for MAIA included ineligibility for ASCT defined by age (≥65 years) or presence of comorbidities precluding high‐dose therapy, Eastern Cooperative Oncology Group (ECOG) performance status ≤2, creatinine clearance ≥30 mL/min, and adequate bone marrow reserve.^2^

#### 2.1.2 Flatiron health database

Records for patients with NDMM were extracted from the Flatiron Health (FH) EHR‐derived deidentified database. The FH database is a nationwide, longitudinal, demographically and geographically diverse database derived from EHR data. The database includes deidentified data from over 280 cancer clinics, primarily community‐based oncology practices, visited by more than 2.4 million US cancer patients. Curated via technology‐enabled abstraction, the FH database includes both structured data (eg, patient demographics, laboratory results, and coded diagnoses) and unstructured data (eg, free text from clinician notes and laboratory reports) from the EHR to define clinical measures that are often unavailable in real‐world data sources, including International Staging System (ISS) stage, ECOG performance status, and disease progression. FH patients were eligible for the present study if they were diagnosed with multiple myeloma (MM) between 1 January 2011 and 30 April 2019 and initiated first‐line therapy (LOT1) at an FH clinic.

#### 2.1.3 Eligibility criteria

Eligibility criteria (Figure S1 in Appendix S1) for the present study included NDMM, age ≥ 65 years, and ineligibility for transplant (MAIA) or no transplant as part of LOT1 (FH). In the FH data, there is no variable reflecting transplant eligibility status; therefore, age ≥ 65 years and no transplant as part of LOT1 were used as a proxy for transplant ineligibility. In addition, the study was restricted to patients who initiated their assigned study regimen (MAIA) or LOT1 (FH) and had ≥1 assessment for disease response following LOT1 initiation. All patients had to meet MAIA trial inclusion criteria, including ECOG performance status ≤2, creatinine clearance ≥30 mL/min, adequate bone marrow reserve and liver function, and absence of selected comorbidities (see Appendix S1). Finally, due to considerations of statistical power, the FH cohort was restricted to LOT1 regimens used by ≥10% of transplant‐ineligible NDMM patients; VRd, Rd, and Vd satisfied this requirement.

#### 2.1.4 Baseline characteristics

Baseline characteristics captured for both MAIA and FH patients included age at LOT1 initiation, sex, race, ISS stage, cytogenetic risk stratification, ECOG performance status, laboratory measures (eg, creatinine clearance, blood counts, liver function enzymes), comorbidities that were MAIA exclusions (eg, severe cardiovascular disease, other primary malignancy), and the time interval from MM diagnosis to the start of LOT1. Details are provided in Appendix S1.

### 2.2 Outcomes

The primary outcome was PFS, defined as the time interval in months between LOT1 initiation and disease progression or death. In the MAIA cohort, the definition of disease progression was based on the International Myeloma Working Group (IMWG) criteria^2^, ^8^, ^9^ for progressive disease. In the FH cohort, a derived progression measure based on the application of IMWG criteria to the setting of real‐world data was used to ascertain progression events. Details on how disease progression and mortality are ascertained in FH are provided in Appendix S1. Patients were followed until their first PFS event, or were censored due to loss of follow‐up or reaching the maximum follow‐up time of 48.5 months (the maximum available for MAIA participants). This analysis includes an additional 9 months of patient follow‐up after the cut‐off date for the first prespecified MAIA interim analysis;^2^, ^10^ details may be found in Appendix S1.

The primary analysis of PFS was an on‐treatment analysis in which patients were also censored if treatment was discontinued for reasons other than disease progression or death. This approach was chosen to reduce heterogeneity in patient management across the MAIA trial and FH routine clinical practice settings. In MAIA, patients were treated until disease progression or unacceptable toxicity. However, this is not always the case in routine clinical practice, where the patient's treatment plan may not include continuous treatment to disease progression, and where patients are more likely to discontinue treatment for a variety of other reasons, including patient preference.^11^ The on‐treatment analysis restricted eligible follow‐up to MAIA and FH patients who did not discontinue their initial treatment regimen; details are provided in Appendix S1. An intent‐to‐treat analysis of PFS, without censoring at treatment discontinuation, was performed as a sensitivity analysis.

Overall survival (OS), defined as the time interval in months between LOT1 initiation and death from any cause, was prespecified as an exploratory endpoint due to the immaturity of the MAIA OS data. Follow‐up for OS in this study was limited to the maximum duration of follow‐up for the first prespecified interim analysis for PFS of MAIA (41.4 months). The next formal analysis of OS in MAIA based on additional follow‐up will occur at the next prespecified interim analysis for OS. Because mortality often occurs after the discontinuation of LOT1 and the initiation of subsequent LOTs, only an intent‐to‐treat analysis was performed for OS.

### 2.3 Statistical methods

The present study used an anchored ITC design (Figure 1), in which relative treatment effects were compared across the MAIA and FH study populations. The methodology follows published guidelines for an anchored matching‐adjusted indirect comparison, a preferred approach for formally comparing results from two trials that share a common comparator or anchor when individual‐level patient data are available from at least one of the trials.^6^, ^7^

An anchored ITC across two distinct study populations requires that the two populations be balanced on treatment effect modifiers. To satisfy this requirement, two steps were taken. First, a common set of eligibility criteria was applied to both the MAIA and FH cohorts. Second, FH patients treated with VRd, Vd, or Rd as LOT1 were weighted to resemble the MAIA trial population on measured baseline characteristics using propensity‐score (PS) methods.^12^ The purpose of the PS weighting was to ensure that (a) patients treated with VRd, Vd, or Rd in FH were balanced on measured baseline covariates, allowing for unconfounded comparisons of outcomes across the FH treatment groups, and (b) to ensure that the FH cohort resembled the MAIA trial population on possible treatment effect modifiers, allowing for a valid ITC to be performed across the two data sources.

Standardized differences were used to assess covariate balance between each of the FH treatment groups and the MAIA trial population after PS weighting. An absolute standardized difference > 0.1 was interpreted as a meaningful covariate imbalance^13^ and was addressed by using both PS weighting and outcome model covariate adjustment (ie, doubly robust estimation).^14^

For the ITC, Rd was used as the common anchor across the MAIA and FH data sources. Within each data source, Cox proportional hazards regression was used to estimate HRs reflecting differences in PFS between treatment groups relative to the common Rd anchor (D‐Rd vs Rd in MAIA; VRd vs Rd and Vd vs Rd in FH). The HRs reflecting direct treatment comparisons within each data source were then used to produce indirect estimates of the HRs for D‐Rd vs VRd and D‐Rd vs Vd.^6^, ^7^ The proportional hazards assumption was evaluated by inspecting the Kaplan‐Meier survival plots and testing the significance of interactions between treatment and follow‐up time.

Multivariate imputation by chained equations was used to impute missing baseline covariate data, and was repeated to create 10 complete datasets.^15^, ^16^ After imputation, analyses were performed separately in each of the 10 complete datasets; the resulting parameter estimates and SEs were pooled according to Rubin's rules to obtain a summary parameter estimate and SE.^17^

All data management and statistical analyses were performed using SAS version 9.4 (SAS Institute, Cary, NC). Additional details and elaboration on the statistical methods including anchored ITC methodology, PS‐weighting, and multiple imputation are provided in Appendix S1.

#### 2.3.1 Subgroup analyses

In a prespecified subgroup analysis, the association between treatment and PFS was assessed in patients aged ≥75 and < 75 years. The subgroup analysis was performed by adding a covariate for age (dichotomized as ≥75 vs <75 years) and an interaction between age and treatment to the Cox regression models.^18^

#### 2.3.2 Sensitivity analyses

Several sensitivity analyses were performed to evaluate the impact of loss to follow‐up and early treatment discontinuation among the FH patients on estimated PFS. First, doubly robust estimation^14^ incorporating both PS‐weighting and regression adjustment for baseline covariates was used; adjusting for baseline covariates in the outcome model mitigates against the possibility of bias due to covariate‐dependent censoring.^19^ Second, we included only FH clinics with an average duration of on‐treatment follow‐up ≥12 months, as the treatment patterns at these clinics would more closely resemble the treat‐to‐progression protocol used in MAIA. Third, we included only patients with ≥12 months of on‐treatment follow‐up. Fourth, the association of treatment with PFS was assessed according to the intent‐to‐treat principle. In this analysis, patients were analyzed according to their initial treatment regimen regardless of subsequent treatment discontinuation.

An additional sensitivity analysis was performed to evaluate the possible impact of multiple imputation of missing baseline covariate data on our results. In this analysis, the FH patients were PS‐weighted based on demographic characteristics alone (age, gender, and race), and clinical characteristics with higher rates of missing data (eg, ISS stage, ECOG performance status) were omitted from the PS model.

#### 2.3.3 Research ethics statement

The MAIA trial protocol was reviewed and approved by an independent ethics committee/institutional review board (IEC/IRB) at all participating sites. All patients participating in the trial provided written informed consent. Retrospective observational research using the deidentified FH database was conducted under an IRB‐approved parent research protocol and waiver of informed consent. Analyses were conducted in accordance with a protocol and statistical analysis plan developed prior to the start of data analysis.

## 3 RESULTS

### 3.1 Baseline patient characteristics

After application of study eligibility criteria, 715 MAIA trial participants (358 D‐Rd, 357 Rd) and 1360 FH NDMM patients (570 VRd, 432 Rd, 358 Vd) were included in the analysis (Figure S1 in Appendix S1). Table 1 shows key baseline characteristics of the MAIA and FH treatment groups after multiple imputation and PS weighting. After weighting, each of the FH treatment groups resembled the MAIA population with respect to age, gender, race, ISS stage, cytogenetic risk stratification, ECOG performance status, creatinine clearance, and time interval between initial MM diagnosis and LOT1 initiation (absolute standardized differences <0.1 for all).

**TABLE 1:** Baseline patient characteristics of MAIA trial participants and Flatiron Health NDMM cohort after multiple imputation and propensity‐score weighting

	MAIA Trial		Flatiron Health NDMM Cohort		
Characteristic	D‐Rd (*n* = 358)	Rd (*n* = 357)	VRd (*n* = 570)	Rd (*n* = 432)	Vd (*n* = 358)
Age in years, mean (SD)	74.2 (5.1)	74.4 (5.4)	74.3 (5.3)	74.3 (5.3)	74.5 (5.6)
Gender, %					
Female	48.9	47.3	48.5	47.6	46.6
Male	51.1	52.7	51.5	52.4	53.4
Race, %					
Black or African American	2.5	4.5	3.4	4.0	3.7
Other	97.5	95.5	96.6	96.0	96.3
ISS Stage, %					
I	27.1	28.3	27.7	28.5	25.6
II	45.5	42.3	43.5	41.0	42.8
III	27.4	29.4	28.8	30.5	31.6
Cytogenetic risk, %					
High risk	15.1	13.9	14.1	14.0	14.8
Standard risk	84.9	86.1	85.9	86.0	85.2
ECOG PS, %					
0	35.2	33.6	35.2	35.0	34.5
1	48.6	50.7	49.1	49.1	48.6
2	16.2	15.7	15.7	15.8	16.9
Creatinine clearance, %					
≤60 mL/min	43.3	38.1	40.6	41.9	42.0
>60 mL/min	56.7	61.9	59.4	58.1	58.0
Months from MM diagnosis to LOT1 start, mean (SD)	1.4 (1.5)	1.3 (1.4)	1.5 (3.6)	1.4 (1.6)	1.3 (2.2)

*Note:* D‐Rd, daratumumab in combination with lenalidomide/dexamethasone; ECOG PS, Eastern Cooperative Oncology Group performance status; ISS, International Staging System; LOT1, first‐line therapy; MM, multiple myeloma; NDMM, newly diagnosed multiple myeloma; Rd, lenalidomide/dexamethasone; SD, standard deviation; Vd, bortezomib in combination with dexamethasone; VRd, bortezomib in combination with lenalidomide/dexamethasone. High cytogenetic risk defined as presence of del17p, t(4;14), or t(14;16) detected based on fluorescence in situ hybridization (FISH); standard risk defined as absence of del17p, t(4;14), or t(14;16) based on FISH. After weighting, each Flatiron Health treatment group resembled the MAIA trial population with respect to each measured baseline covariate (absolute standardized differences <0.1).

### 3.2 Imputation of missing baseline covariate data

In FH, the percentage of patients with missing data was low for race (8%), ranged from 13%‐27% for the lab results with the exception of neutrophil count (55%), and was 30%, 40%, and 47% for cytogenetic risk, ECOG PS, and ISS stage, respectively. Data were complete for other baseline covariates in FH. In MAIA, data were missing on cytogenetic risk for 13% of patients; data were complete for other baseline covariates. For all covariates with missing values, covariate distributions were similar before and after multiple imputation (Tables S2 and S3 in Appendix S1).

### 3.3 Primary outcome: Progression‐free survival

In MAIA, D‐Rd was associated with a significantly lower risk of disease progression or death compared to Rd over a mean follow‐up for 24.9 months (HR 0.54; 95% CI 0.42, 0.71; *P* < .001; Figure 1). Note that, due to the exclusion of 22 MAIA patients (Figure S1 in Appendix S1), the censoring of patients who discontinued treatment for reasons other than progression or death, and the inclusion of an additional 9 months of follow‐up data, this point estimate differs slightly from the HR of 0.56 reported in the first interim analysis of the MAIA study by Facon et al^2^ Please see Appendix S1 for a full explanation.

**FIGURE 2:**
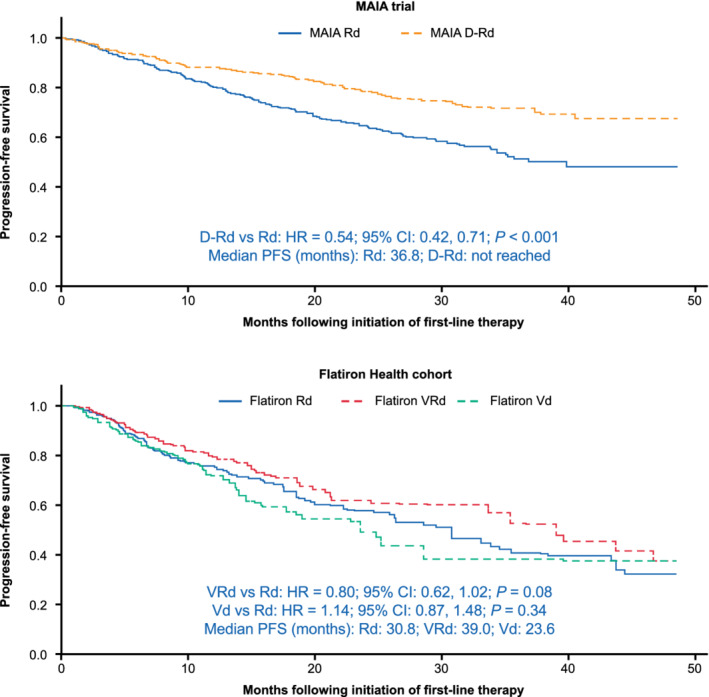
Within‐study comparisons of progression‐free survival in MAIA trial and Flatiron Health patient cohorts by first‐line treatment regimen. CI, confidence interval; D‐Rd, daratumumab‐lenalidomide‐dexamethasone; HR, hazard ratio; Rd, lenalidomide‐dexamethasone; Vd, bortezomib‐dexamethasone; VRd, bortezomib‐lenalidomide‐dexamethasone. Figure reflects results for primary on‐treatment analysis of progression‐free survival with weighting of Flatiron Health treatment groups to resemble MAIA trial population [Color figure can be viewed at wileyonlinelibrary.com]

In FH, VRd was associated with a non‐significantly lower risk of disease progression or death relative to Rd (HR 0.80; 95% CI 0.62, 1.02; *P* = .08), and Vd with a non‐significantly higher risk of disease progression or death relative to Rd over a mean follow‐up of 10.9 months (HR 1.14; 95% CI 0.87, 1.48; *P* = .34; Figure 2). Details on the duration of follow‐up and reasons for censoring for each data source are provided in Appendix S1. No evidence for a violation of the proportional hazards assumption was found in MAIA or FH. The anchored ITC across MAIA and FH based on these effect estimates showed that D‐Rd was associated with a significantly lower risk of disease progression or death relative to VRd (HR 0.68; 95% CI 0.48, 0.98; *P* = .04) and Vd (HR 0.48; 95% CI 0.33, 0.69; *P* < .001; Figure 1).

For the subgroup analysis based on age, doubly robust estimation^14^ was used to account for the fact that absolute standardized differences for some baseline covariates were ≥ 0.1 within strata defined by age (≥75 and < 75 years). In the age subgroup analysis, there was no evidence of significant interaction between age and any of the treatment comparisons of interest (*P* = .24 for D‐Rd vs VRd, *P* = .70 for D‐Rd vs Vd, and *P* = .97 for D‐Rd vs Rd). The D‐Rd vs VRd contrast was smaller in magnitude in patients aged ≥75 years (HR 0.80; 95% CI 0.51, 1.26; *P* = .33) than in patients aged <75 years (HR 0.58, 95% CI 0.37, 0.91; *P* = .02); however, the difference between these effect estimates was not statistically significant (*P* = .24; Table 2).

**TABLE 2:** Summary of primary, sensitivity, and subgroup analyses of progression‐free survival

	Direct within‐study treatment comparisons			Indirect treatment comparisons	
	MAIA D‐Rd vs MAIA Rd	FH VRd vs FH Rd	FH Vd vs FH Rd	MAIA D‐Rd vs FH VRd	MAIA D‐Rd vs FH Vd
Primary analysis: On‐treatment analysis of PFS	0.54 (0.42, 0.71), *P* < .001	0.80 (0.62, 1.02), *P* = .08	1.14 (0.87, 1.48), *P* = .34	0.68 (0.48, 0.98), *P* = .04	0.48 (0.33, 0.69), *P* < .001
Sensitivity analyses					
Adjustment for demographics only	0.54 (0.42, 0.71), *P* < .001	0.82 (0.64, 1.05), *P* = .11	1.24 (0.97, 1.57), *P* = .08	0.66 (0.46, 0.95), *P* = .02	0.44 (0.31, 0.63), *P* < .001
Doubly robust estimation of association between treatment and PFS	0.54 (0.42, 0.71), *P* < .001	0.79 (0.61, 1.01), *P* = .06	1.11 (0.84, 1.48), *P* = .44	0.69 (0.48, 0.99), *P* = .045	0.49 (0.33, 0.71), *P* < .001
Restrict to FH clinics with average on‐treatment follow‐up ≥12 mo	0.54 (0.42, 0.71), *P* < .001	0.81 (0.51, 1.28), *P* = .36	1.22 (0.66, 2.27), *P* = .51	0.67 (0.23, 0.85), *P* = .14	0.45 (0.23, 0.85), *P* = .01
Restrict to patients with ≥12 mo on‐treatment follow‐up	0.50 (0.35, 0.71), *P* < .001	0.73 (0.49, 1.10), *P* = .14	1.23 (0.71, 2.12), *P* = .46	0.69 (0.40, 1.17), *P* = .17	0.41 (0.22, 0.78), *P* = .006
Intent‐to‐treat analysis	0.53 (0.42, 0.67), *P* < .001	1.02 (0.84, 1.25), *P* = .83	1.35 (1.09, 1.68), *P* = .006	0.52 (0.38, 0.70), *P* < .001	0.39 (0.28, 0.54), *P* < .001
Subgroup analyses					
≥75 y of age	0.55 (0.40, 0.79), *P* = .001	0.69 (0.49, 0.97), *P* = .03	1.08 (0.79, 1.47), *P* = .64	0.80 (0.51, 1.26), *P* = .33	0.51 (0.33, 0.79), *P* = .002
<75 y of age	0.55 (0.39, 0.78), *P* = .001	0.94 (0.67, 1.32), *P* = .72	1.18 (0.81, 1.72), *P* = .37	0.58 (0.37, 0.91), *P* = .02	0.46 (0.28, 0.76), *P* = .03

*Note:* Data are HR estimates followed by 95% confidence intervals in parentheses. D‐Rd, daratumumab in combination with lenalidomide/dexamethasone; FH, Flatiron Health; HR, hazard ratio; PFS, progression‐free survival; Rd, lenalidomide/dexamethasone; Vd, bortezomib in combination with dexamethasone; VRd, bortezomib in combination with lenalidomide/dexamethasone. Please note that the following analyses were based on subgroups of the total eligible sample (n = 2075): restricting to patients with ≥12 mo on‐treatment follow‐up (n = 910); restricting to Flatiron Health clinics with average on‐treatment follow‐up ≥12 mo (n = 1115); age ≥ 75 y (n = 1043); age < 75 y (n = 1032). These analyses had less statistical power because they were not based on the full sample.

### 3.4 Sensitivity analyses

Table 2 provides a summary of the results from sensitivity analyses. In the sensitivity analysis of PFS, which adjusted for only demographic characteristics (age, sex, and race), results were similar to the primary PFS analyses. Results from additional sensitivity analyses that assessed the possible impact of treatment discontinuation prior to disease progression and loss to follow‐up were consistent with the principal study findings. In the intent‐to‐treat sensitivity analysis, the difference in PFS between FH patients treated with VRd and Rd was smaller in magnitude, which likely reflects higher rates of treatment discontinuation among patients treated with VRd in routine clinical practice (Figure S2 in Appendix S1).

### 3.5 Exploratory outcome: Overall survival

Formal ITCs across data sources for OS were not conducted because the MAIA OS data remain immature, with median OS reached in neither trial arm. Follow‐up for OS in MAIA is ongoing. Based on the MAIA trial data, the HR for OS associated with D‐Rd was 0.73 (95% CI 0.52, 1.04) relative to Rd over a mean follow‐up of 25.3 months. In FH, relative to Rd, the OS HRs for VRd and Vd were 0.88 (95% CI 0.69, 1.11) and 1.24 (95% CI 1.00, 1.55), respectively, over a mean follow‐up of 26.1 months (Figure S3 in Appendix S1).

## 4 DISCUSSION

No clinical trials have directly compared D‐Rd to SOC regimens other than Rd in patients with transplant‐ineligible NDMM;^2^ the present study was conducted to address this evidence gap. Based on the Rd‐anchored ITC, treatment with D‐Rd was associated with a significant 32% reduced risk of disease progression or death relative to VRd, and with a significant 52% reduced risk of disease progression or death relative to Vd in patients with transplant‐ineligible NDMM. This study used well‐established ITC methods in a novel approach to incorporate both clinical trial data and real‐world data to address an important clinical question that at present cannot be addressed with either data source alone. In the absence of head‐to‐head clinical trials comparing these regimens, these findings provide insight into the PFS benefit of the newly approved D‐Rd treatment combination regimen over other commonly used SOC regimens for transplant‐ineligible NDMM patients.

The direct treatment comparisons from the FH patient cohort should be put in context of relevant clinical trial data. In FH, treatment with VRd was associated with a 20% lower risk of disease progression or death compared to Rd (HR 0.80; 95% CI 0.62, 1.02; *P =* .08). In the SWOG S0777 trial, which compared VRd and Rd treatment in patients with NDMM without intent for immediate ASCT, treatment with VRd was associated with a 26% lower risk of disease progression or death compared to Rd (HR 0.74; 96% CI 0.59, 0.93; *P =* .003) based on the most recent dataset update.^20^ The difference in the HR estimates for VRd vs Rd in the present study and vs SWOG S0777 may be attributable to sampling variation (the CIs for the HRs have considerable overlap) and differences in the patient populations. In SWOG S0777, 69% of participants had intent for an eventual transplant at disease progression, and the median age of patients was 63 vs 74 years in the PEGASUS study. To our knowledge, PFS HRs for VRd vs Rd in SWOG S0777 have not been published for subgroups of patients aged <65 and ≥ 65 years. However, in subgroup analyses of OS based on age presented in the most recent SWOG S0777 update,^20^ HRs for VRd vs Rd were 0.64 (*P* = .028) and 0.77 (*P* = .168) for patients aged <65 and ≥ 65 years, respectively, suggesting that there may be clinically meaningful treatment heterogeneity based on age and fitness, with a smaller relative treatment effect for VRd vs Rd observed in patients ≥65 years old.

Regarding the comparison of Vd and Rd in FH, these regimens have not been directly compared in a head‐to‐head trial. However, a recent network meta‐analysis that compared PFS associated with different treatment regimens in the setting of transplant‐ineligible NDMM suggested that Vd and Rd are not significantly different in terms of treatment effectiveness,^21^ which is consistent with our finding of no statistically significant differences between Vd and Rd in FH.

Use of data from both clinical trial and routine clinical practice settings in this study presented several challenges. Clinical trial participants are often healthier than typical patients in routine clinical practice. While we excluded FH patients with documented comorbidities constituting MAIA trial exclusions (Figure S1 in Appendix S1), it is possible that these conditions may be under‐reported and/or under‐coded in the structured FH EHR data, and thus that the included FH cohort may have had a greater comorbidity burden than the MAIA trial participants. In addition, it was not possible to assess comorbidity severity using the structured diagnosis codes available in the FH EHR data. However, as discussed in greater detail below, the anchored ITC design means that the possibility of unmeasured differences in comorbidity burden across the two populations will only cause bias if the unmeasured comorbidities are relative treatment effect modifiers.

Patient management is also likely to differ in trial and real‐world settings.^11^ Patients in MAIA were treated with D‐Rd or Rd until disease progression or unacceptable toxicity. Patients identified from the FH database were treated in real‐world clinical settings according to the judgment of their treating physician rather than a protocol‐driven standardized treatment plan. In addition, medication management to maximize treatment persistence may be more optimal in clinical trial settings. To account for this inconsistency, the primary analyses in this study were on‐treatment analyses, with patients censored at treatment discontinuation for reasons other than disease progression or death. The on‐treatment analysis and the anchored ITC study design help to minimize the impact of differences in patient management and treatment duration in the MAIA trial vs routine clinical practice, where treatment discontinuation in the absence of disease progression or death may be more common. Sensitivity analyses that explored the possible impact of treatment discontinuation and loss to follow‐up among FH patients resulted in findings that were similar to the principal study findings (Table 2). In addition, dosing and treatment schedules may vary in real‐world settings compared with clinical trials. However, data on dosing were limited in FH. The anchored ITC study design would mitigate against bias due to systemic differences in dose intensity in real‐world vs clinical trial settings.

In addition, methods for assessing progression varied for the study populations, with MAIA trial patients having disease progression assessed at prespecified time intervals based on IMWG criteria.^2^, ^8^, ^9^ In contrast, FH patients may not have been assessed for progression at regular intervals, and, as detailed in Appendix S1, some IMWG criteria for progression, such as development of plasmacytoma, are not included in FH's disease progression algorithm, which considers only laboratory M‐protein and free light chain measurements. This systematic difference in outcome event ascertainment was one of the reasons for performing an anchored ITC, in which there was no direct comparison of absolute outcome event rates across the MAIA and FH patient populations. Since only relative treatment effects are compared across data sources, the anchored ITC mitigates the risk of bias due to differences in outcome event ascertainment across data sources. (For example, a relative risk estimate *r*_*a*_/*r*_*b*_ comparing groups A and B remains unbiased if only 90% of outcome events are ascertained in group A and group B.)

Due to these differences in patient populations, care settings, and methods for outcome ascertainment across MAIA and FH, an anchored ITC study design was chosen. An anchored ITC requires that the two populations be balanced on relevant effect modifiers but does *not* require (a) that the populations are the same with respect to all prognostic factors, or (b) that the absolute outcome event rates in the treatment group serving as the common anchor are identical in the two data sources.^6^, ^7^ As evidenced by the difference in PFS between the MAIA Rd and FH Rd patients (Figure 1), even after efforts to balance the patient populations there may still be unmeasured or residual differences in prognostic factors between the trial and real‐world patient populations. The anchored ITC design protects against this source of bias by comparing treatment effects relative to the Rd anchor rather than directly comparing outcome rates across the data sources. Within FH, however, comparisons among patients who received VRd, Rd, or Vd may be subject to bias due to confounding by differences in baseline prognostic factors; confounding was addressed through PS weighting and in a sensitivity analysis with doubly robust estimation, which resulted in similar effect estimates.

There were missing data on key patient clinical characteristics among the study groups identified from the FH database. An explicit determination of transplant eligibility status was unavailable in the FH database; therefore, age ≥ 65 years and no receipt of transplant during LOT1 were used as a proxy for transplant ineligibility. For consistency, the age ≥ 65 restriction was also applied to the MAIA trial population. Multiple imputation was employed to account for other missing baseline covariate data; however, the validity of multiple imputation relies on the assumption that the data are missing at random (MAR)—ie, that the probability of a data point being missing is unrelated to the true value of that missing data point after conditioning on the variables included in the imputation models.^15^ While the MAR assumption cannot be proven, the fact that the study results were consistent in the sensitivity analysis where only patients' demographics were adjusted for provides reassurance that the results were not sensitive to the imputation of the missing clinical characteristics.

Based on the MAIA trial data, D‐Rd treatment in patients with transplant‐ineligible NDMM was associated with a 46% significantly lower risk of disease progression or death relative to Rd alone. Based on the Rd‐anchored ITCs performed in the present study, D‐Rd treatment was associated with a 32% and 52% significantly lower risk of disease progression or death compared to the alternative SOC regimens VRd and Vd, respectively. In the absence of head‐to‐head trial data, the present ITC may help to inform treatment selection in patients with transplant‐ineligible NDMM.

## CONFLICT OF INTEREST

BD ‐ Consultancy: Amgen, Celgene, Johnson & Johnson, and Takeda. SK ‐ Takeda: Research Funding: Takeda, Janssen, Celgene; Consultancy: Janssen, Celgene. SU ‐ Speakers bureau: Amgen, Celgene, Janssen, Sanofi, Takeda; Research Funding: Amgen Array Biopharma, Bristol‐Myers Squibb, Celgene, Janssen, Merck, Pharmacyclics, Sanofi, Takeda; Consultancy: Amgen, Bristol‐Myers Squibb, Celgene, Janssen, Merck, SkylineDx, Takeda. EA, AL, and RK ‐ Employment and equity: Janssen. EM was employed by Janssen when the study was conducted. TF ‐ Advisory committee: Celgene, Janssen, Takeda, Amgen, Sanofi; Speakers bureau: Celgene, Janssen, Takeda.

## DATA AVAILABILITY

Requests for access to the MAIA trial study data may be submitted through the Yale Open Data Access (YODA) Project site at http://yoda.yale.edu. The data sharing policy of Janssen Pharmaceutical Companies is available at https://www.janssen.com/clinical-trials/transparency. The deidentified data originating from Flatiron Health, Inc., may be made available upon request, and are subject to a license agreement with Flatiron Health; interested researchers should contact DataAccess@FH.com to determine licensing terms.

## Supporting information


**Appendix** **S1**: Supporting Information.
