# Supplementary material for: Daratumumab‐lenalidomide‐dexamethasone vs standard‐of‐care regimens: Efficacy in transplant‐ineligible untreated myeloma
Source: Am J Hematol. 2020 Sep 5;95(12):1486–94. doi: 10.1002/ajh.25963 (PMC7754114; doi:10.1002/ajh.25963)
Supplement: Supplementary file 1 — Appendix S1: Supporting Information. [file AJH-95-1486-s001.docx]

Contents

[Methods: MAIA trial exclusion criteria applied to Flatiron Health cohort. 2](#_Toc46488436)

[Methods: Ascertainment of disease progression and mortality in the Flatiron Health cohort. 3](#_Toc46488437)

[Methods: Eligible study population and follow-up for progression-free survival for MAIA patients in present study and prior MAIA trial publications. 4](#_Toc46488438)

[Supplemental Table 1. Progression-free survival D-Rd vs Rd estimates reported in present study and prior MAIA trial reports. 5](#_Toc46488439)

[Methods: On-treatment and intent-to-treat analyses of progression-free survival. 6](#_Toc46488440)

[Methods: Anchored indirect treatment comparison study design. 7](#_Toc46488441)

[Methods: Details on propensity-score weighting 9](#_Toc46488442)

[Methods: Multiple imputation of missing baseline covariate data. 9](#_Toc46488443)

[Supplemental Table 2. Details on degree of missingness for each variable in MAIA and Flatiron Health (FH) and how each variable was used in the analysis. 11](#_Toc46488444)

[Supplemental Table 3 Summary of baseline covariate degree of missingness and distributions before and after multiple imputation (MI) for the Flatiron Health (FH) newly diagnosed multiple myeloma cohort (n = 1957). 13](#_Toc46488445)

[Results: Details on censoring and loss to follow-up 15](#_Toc46488446)

[Supplemental Table 4 Reasons for end of follow-up in MAIA and Flatiron Health patient cohorts. 15](#_Toc46488447)

[Supplemental Figure 1 Selection of eligible MAIA trial participants and Flatiron Health patients with transplant-ineligible newly diagnosed multiple myeloma. 16](#_Toc46488448)

[Supplemental Figure 2 Progression-free survival and loss to follow-up in the primary on-treatment analysis of PFS in the MAIA trial and Flatiron Health patient cohorts by first-line treatment regimen. 17](#_Toc46488449)

[Supplemental Figure 3 Overall survival hazard ratios for D-Rd relative to alternative standard-of-care regimens based on direct and indirect treatment comparisons. 18](#_Toc46488450)

[References 19](#_Toc46488451)

# Methods: MAIA trial exclusion criteria applied to Flatiron Health cohort.

As shown in Figure 1, after the selection of newly diagnosed multiple myeloma (NDMM) patients aged ≥65 years who did not undergo stem cell transplantation and who received bortezomib, lenalidomide, and dexamethasone (VRd), bortezomib and dexamethasone (Vd), or lenalidomide and dexamethasone (Rd) as first line of therapy (LOT1) from the Flatiron Health (FH) database, additional MAIA exclusion criteria were applied to the FH cohort. Below are additional details on these exclusions.

- Creatinine clearance <30 mL/min.
- Eastern Cooperative Oncology Group (ECOG) Performance Status (PS) score >2.
- Laboratory values indicative of significant impairment in bone marrow reserve or liver function or of hypercalcemia. Specifically, patients with measures *outside* the following ranges were excluded:
  - Hemoglobin ≥7.5 g/dL
  - Absolute neutrophil count ≥1.0 x 10^9^/L
  - Platelet count ≥70 x 10^9^/L
  - Aspartate aminotransferase ≤2.5 x upper limit of normal (ULN);
  - Alanine aminotransferase ≤2.5 x ULN;
  - Total bilirubin ≤2.0 x ULN;
  - Corrected serum calcium ≤14 mg/dL or free ionized calcium ≤6.5 mg/dL
- The presence of selected comorbidities or recent medical events:
  - Acute myocardial infarction
  - Congestive heart failure
  - Chronic obstructive pulmonary disease
  - Asthma
  - Human immunodeficiency virus
  - Hepatitis
  - Active systemic infection
  - Alcoholism, substance abuse disorder, or major psychiatric or neurologic disorder
  - Primary malignancy other than multiple myeloma (excluding treatable skin and cervical cancers)

# Methods: Ascertainment of disease progression and mortality in the Flatiron Health cohort.

In the FH cohort, disease progression status is derived from laboratory measures of myeloma disease burden. First, each patient at treatment initiation is assigned a biomarker for tracking disease progression:

- First choice: serum M-protein measure. Must have measurable disease at baseline (serum M-protein ≥1 g/dL) to use serum M-protein for tracking progression.
- Second choice: urine M-protein measure. Must have measurable disease at baseline (urine M-protein ≥200 mg) to use urine M-protein for tracking progression.
- Third choice: free light chain (FLC) ratio.

Once a baseline biomarker for tracking disease progression has been defined, the patient is tracked longitudinally for a clinically meaningful increase in serum M-protein, urine M-protein, or the FLC ratio, based on International Myeloma Working Group (IMWG) criteria.[1, 2] However, it should be noted that the FH-derived progression algorithm does not consider the following IMWG criteria in ascertaining progression events:

- ≥ 25% increase in bone marrow plasma cell percentage from nadir and absolute increase ≥10%.
- Definite development of new bone lesions or soft tissue plasmacytomas or definite increase in the size of existing bone lesions or soft tissue plasmacytomas.
- Development of hypercalcemia that can be attributed solely to the plasma cell proliferative disorder.

These three IMWG criteria for disease progression were considered in defining progression events in the MAIA trial, but are not considered in the FH-derived progression algorithm. This systematic difference in outcome event ascertainment was one of the reasons for performing an indirect treatment comparison in which there are no direct comparisons of outcome event rates across the MAIA and FH patient populations.

With respect to the ascertainment of mortality, the FH mortality data are obtained from several sources, including the EHR, external commercial data sources that mine data from obituaries, funeral homes, and other sources, and data from the US Social Security Death Index. FH has benchmarked its mortality data against the US National Death Index, and reports that its composite measure of mortality has a 97–98% positive predictive value and 85–90% sensitivity when compared against the gold-standard National Death Index.[3]

# Methods: Eligible study population and follow-up for progression-free survival for MAIA patients in present study and prior MAIA trial publications.

The present study excluded MAIA trial participants aged <65 years (n = 8), who had ECOG PS >2 (n = 2), who did not initiate study treatment (n = 9), or who did not have ≥1 assessment for disease progression following LOT1 initiation (n = 3; see Figure 1). Note that because the present study excluded a total of 22 MAIA trial participants and was an on-treatment analysis (rather than intent-to-treat), the D-Rd vs. Rd hazard ratio (HR) for progression-free survival (PFS) in the present study differs slightly from what was reported in the recent MAIA trial update (Supplemental Table 1). Both the present study and the MAIA trial update included ~9 months of additional follow-up for PFS after the first interim analysis.

## Table S1. Progression-free survival D-Rd vs Rd estimates reported in present study and prior MAIA trial reports.

| **Analysis** | **MAIA D-Rd vs. Rd**  **HR (95% CI)** |
| --- | --- |
| First interim analysis of MAIA[4] | 0.56 (0.43, 0.73) |
| MAIA update with ~9 months additional follow-up for PFS[5] | 0.56 (0.44, 0.71) |
| Present PEGASUS study, on-treatment analysis based on the MAIA update with ~9 months additional follow-up for PFS | 0.54 (0.42, 0.71) |
| Present PEGASUS study, intent-to-treat analysis based on the MAIA update with ~9 months additional follow-up for PFS | 0.53 (0.42, 0.67) |

# Methods: On-treatment and intent-to-treat analyses of progression-free survival.

The primary analysis of PFS was an on-treatment analysis in which patients were censored if treatment was discontinued for reasons other than disease progression or death. This approach was chosen to reduce heterogeneity in patient management across the MAIA trial and FH routine clinical practice settings. In the MAIA trial, patients were treated until disease progression or unacceptable toxicity. However, this is not always the case in routine clinical practice, where the patient’s treatment plan may not include continuous treatment to disease progression, and where patients may discontinue treatment for a variety of other reasons, including patient preferences.[6] The on-treatment analysis restricted eligible follow-up to MAIA and FH patients who did not discontinue their initial treatment regimen. Treatment discontinuation was operationally defined as a gap in antimyeloma therapy ≥90 days (ignoring systemic corticosteroid use), as specified below:

- For VRd patients: ≥90 days with no V use and no R use. In addition, patient was censored if either V or R was discontinued in 90 days after initiation of LOT1.
- For D-Rd patients: ≥90 days with no D use and no R use. In addition, patient was censored if either D or R was discontinued in 90 days after initiation of LOT1.
- For Vd patients: ≥90 days with no use of V.
- For Rd patients: ≥90 days with no use of R.

In the intent-to-treat sensitivity analysis of PFS, patients were not censored for treatment discontinuation. In both the primary on-treatment analysis and sensitivity intent-to-treat analysis of PFS, patients were censored if they were lost to follow-up for disease progression, initiated second-line therapy (following the MAIA trial’s rules for follow-up for PFS), or reached the dataset cut-off date. In the FH cohort, the mean duration of follow-up for PFS was 10.9 months in the on-treatment analyses and 13.8 months in the intent-to-treat analysis.

# Methods: Anchored indirect treatment comparison study design.

The present study used an anchored indirect treatment comparison (ITC) study design (Figure 2), which involves a comparison of relative treatment effects across two distinct study populations. The methodology follows published guidelines for an anchored matching-adjusted indirect comparison, a preferred approach for formally comparing results from two trials that share a common comparator or anchor when individual-level patient data are available from at least one of the trials.[7, 8] An anchored ITC across two distinct study populations requires that the two populations be balanced on treatment effect modifiers. To satisfy this requirement, two steps were taken. First, a common set of inclusion criteria were applied to both the MAIA and FH cohorts; second, the FH patients treated with VRd, Vd, or Rd as LOT1 were weighted to resemble the MAIA trial population on measured baseline characteristics using propensity score (PS) weighting.[9] The purpose of the PS weighting was to ensure that (1) patients treated with VRd, Vd, or Rd in FH were similar on measured baseline covariates, allowing for unconfounded comparisons of outcomes across the FH treatment groups, and (2) to ensure that the FH cohort resembled the MAIA trial population on relevant effect modifiers, allowing for a valid ITC to be performed across the two data sources.

Standardized differences were used to assess covariate balance between each of the FH treatment groups and the MAIA trial population after PS weighting. A standardized difference >0.1 was interpreted as meaningful covariate imbalance[10] and was addressed by using both PS weighting and outcome model covariate adjustment (i.e., doubly robust estimation).[11]

For the ITC, Rd was used as the common anchor across the MAIA and FH data sources. Within each data source, Cox proportional hazards regression was used to estimate HRs reflecting differences in PFS between treatment groups relative to the common Rd anchor (D-Rd vs Rd in MAIA; VRd vs Rd and Vd vs Rd in FH). The HRs reflecting direct treatment comparisons within each data source were then compared to produce indirect estimates of the HRs for D-Rd vs VRd and D-Rd vs Vd.[7, 8]

An anchored ITC involves a contrast of relative treatment effects (here, HRs) estimated in two distinct trials or study populations that share a common treatment group or anchor. In MAIA, D-Rd was compared with Rd; in FH, VRd and Vd were each compared to Rd. The ln(HRs) reflecting these direct treatment comparisons were subtracted to obtain indirect estimates of the effectiveness of D-Rd vs VRd and D-Rd vs Vd, as shown below.

$$\ln\left( HR_{D-Rd vs VRd} \right)=\ln\left( HR_{D-Rd vs Rd} \right)-\ln\left( HR_{VRd vs Rd} \right)$$

$$\ln\left( HR_{D-Rd vs Vd} \right)=\ln\left( HR_{D-Rd vs Rd} \right)-\ln\left( HR_{Vd vs Rd} \right)$$

Because the MAIA and FH samples are independent, for each indirect ln(HR) estimate, the variance was calculated as the sum of the estimated variances for the two ln(HR) point estimates being contrasted, as shown below.

$$var_{\ln\left( HR_{D-Rd vs. VRd} \right)}=var_{\ln\left( HR_{D-Rd vs. Rd} \right)}+var_{\ln\left( HR_{VRd vs. Rd} \right)}$$

$$var_{\ln\left( HR_{D-Rd vs. Vd} \right)}=var_{\ln\left( HR_{D-Rd vs. Rd} \right)}+var_{\ln\left( HR_{Vd vs. Rd} \right)}$$

The rationale for using an anchored ITC study design is that it makes fewer assumptions than a direct treatment comparison across different study populations. In contrast to a direct comparison of treatment groups across data sources, an anchored ITC requires that the MAIA and FH patient populations be balanced on relevant effect modifiers, but does *not* require the populations to be balanced on all prognostic factors (i.e., confounders).[7, 8] The use of Rd as a common anchor for indirectly comparing D-Rd and real-world standard-of-care treatment regimens allows us to appropriately account for the possibility of unmeasured or residual differences in prognostic factors between the MAIA trial and FH routine clinical practice patient populations that remain after the application of study inclusion criteria and PS weighting.

# Methods: Details on propensity-score weighting

As discussed above, PS weighting was used in the present study to address potential confounding within FH and to balance the FH and MAIA patient populations on possible treatment effect modifiers. A separate PS model and weighting scheme was developed for each real-world treatment group of interest (VRd, Vd, and Rd). After fitting each PS model using logistic regression, the following weighting scheme was applied: each MAIA patient received a weight of 1, and each FH patient received a weight of (PS/(1-PS)), where PS was the estimated propensity score for a given patient.[9] With these weights applied, each of the FH treatment groups (VRd, Vd, and Rd) would be expected to resemble the MAIA patient population with respect to measured baseline characteristics. Weighting in this manner is a standard technique for statistical adjustment in ITCs and more broadly for causal inference in the biomedical and social sciences, and is analogous to the use of weighting to account for under-representation or over-representation of particular population subgroups in survey research due to non-random sampling and/or non-response.[12]

# Methods: Multiple imputation of missing baseline covariate data.

Missing data were addressed with multiple imputation.[3, 13] Imputation of missing data was done using multivariate imputation by chained equations,[3] and was repeated to create 10 complete datasets. Predictive mean matching was used to impute continuous and ordinal variables, logistic regression was used to impute binary variables, and multinomial logistic regression was used to impute polytomous categorical variables.[13] The imputations were done with PROC MI in SAS 9.4.

Imputation of missing data was performed separately for each data source and treatment group (MAIA D-Rd, MAIA Rd, FH VRd, FH Rd, FH Vd). This approach is equivalent to including data source and treatment group in the prediction models as well as interactions between these classifications and the other variables included in the imputation models. Supplemental Table 2 shows the degree of missingness for each baseline covariate in FH and MAIA, the variables included in each imputation model, whether the variable was used as a study inclusion/exclusion criterion, and whether the variable was included in the propensity-score model that was used to weight the FH patients to resemble the MAIA trial population. In FH, a number of baseline covariates had some degree of missing data (see Supplemental Table 2 and Supplemental Table 3); in MAIA, there was missing data only for cytogenetic risk for 12.7% of patients.

Because data were missing in FH for some of the baseline covariates constituting MAIA trial exclusion criteria (eg, creatinine clearance, blood counts, ECOG PS), imputation of missing data was performed *prior* to the application of these MAIA exclusion criteria to the FH cohort. Thus, the patient sample in which the imputation models were fit included 1957 FH non-transplant NDMM patients aged 65+ years who initiated VRd, Rd or Vd as first-line therapy (see Figure 1, patient selection flow diagram), after which the MAIA trial inclusion criteria were applied (creatinine clearance ≥30 mL/min, ECOG PS ≤2, adequate bone marrow reserve and liver function, absence of selected comorbidities).

## Table S2. Details on degree of missingness for each variable in MAIA and Flatiron Health (FH) and how each variable was used in the analysis.

| **Variable type** | **Variable** | **Inclusion/ exclusion criteria** | **Included in PS model** | **Missing % in FH** | **FH imputation model** | **Missing % in MAIA** | **MAIA imputation model** |
| --- | --- | --- | --- | --- | --- | --- | --- |
| Demographic | Age in years at LOT1 initiation (continuous) | Y | Y | 0.0% | Y | 0.0% | Y |
|  | Sex (binary: female; male) | N | Y | 0.0% | Y | 0.0% | Y |
|  | Race (binary: black or African-American; other) | N | Y | 8.1% | Y | 0.0% | Y |
| Clinical | International Staging System (ISS) Stage (ordinal: I; II; III) | N | Y | 47.2% | Y | 0.0% | Y |
|  | Cytogenetic risk stratification (categorical: high risk; standard risk) | N | Y | 29.6% | Y | 12.7% | Y |
|  | ECOG PS (ordinal: 0; 1; 2; 3; 4) | Y | Y | 39.5% | Y | 0.0% | Y |
|  | Months from MM diagnosis to LOT1 start (continuous) | N | Y | 0.0% | Y | 0.0% | Y |
| Labs | Creatinine clearance (continuous for imputation; dichotomized as ≤60 vs. >60 for PS-weighting) | Y | Y | 13.5% | Y | 0.0% | Y |
|  | Hemoglobin (continuous) | Y | N | 13.0% | Y | 0.0% | N |
|  | Platelet count (continuous) | Y | N | 26.5% | Y | 0.0% | N |
|  | Neutrophil count (continuous) | Y | N | 54.6% | Y | 0.0% | N |
|  | Corrected serum calcium (continuous) | Y | N | 19.4% | Y | 0.0% | N |
|  | Serum albumin (continuous) | Y | N | 24.5% | Y | 0.0% | N |
|  | Total bilirubin (continuous) | Y | N | 22.5% | Y | 0.0% | N |
|  | Serum aspartate aminotransferase | Y | N | 20.1% | Y | 0.0% | N |
|  | Serum alanine aminotransferase | Y | N | 21.2% | Y | 0.0% | N |
| Year | Calendar year at LOT1 initiation | N | N | 0.0% | Y | 0.0% | N |
| Vitals | Body mass index | N | N | 8.0% | Y | 0.0% | N |
| Outcomes | Progression-free survival (indicator for PFS event vs. censoring; continuous time-to-event variable; interaction term between the two terms) | N | N | 0.0% | Y | 0.0% | N |
|  | Overall survival (indicator for OS event vs. censoring; continuous time-to-event variable; interaction term between the two terms) | N | N | 0.0% | Y | 0.0% | N |

Y indicates yes; N, no. ECOG PS, Eastern Cooperative Oncology Group performance status; LOT1, first-line therapy; MM, multiple myeloma; PFS, progression-free survival; PS, propensity score. High risk cytogenetics defined as presence of del17p, t(4;14), or t(14;16) detected based on fluorescence in situ hybridization (FISH); standard risk defined as absence of del17p, t(4;14), or t(14;16) based on FISH. Columns indicate whether the covariate was used for study inclusion criteria, included in PS model used to weight FH patients, degree of missing data in FH, whether the covariate was included in imputation models for the FH cohort, degree of missing data in MAIA, and whether the covariate was included in imputation models for the MAIA cohort.

Supplemental Table 3 provides details on the degree of missingness for each baseline covariate and the baseline covariate distributions before and after multiple imputation in FH. There were no missing data for age, sex, calendar year, time from multiple myeloma diagnosis to treatment initiation, and the outcome variables. The proportion of FH patients with missing data was low for race (8%), ranged from 13–27% for the lab results with the exception of neutrophil count (55%), and was 30%, 40%, and 47% for cytogenetic risk, ECOG PS, and ISS stage, respectively. For all covariates with missing values, covariate distributions in FH were similar before and after multiple imputation (Supplemental Table 3).

In MAIA, data were missing only for cytogenetic risk for 12.7% of patients. Prior to imputation, 14.3% of MAIA patients were classified as having high-risk cytogenetics; after imputation, 14.6% were classified as having high-risk cytogenetics.

After imputation, analyses were performed separately in each of the 10 complete datasets; the resulting parameter estimates and standard errors were pooled according to Rubin’s rules to obtain a summary parameter estimate and standard error reflecting both the within- and between-imputation variance associated with the parameter estimate.[13] The pooling of parameter estimates was done with PROC MIANALYZE in SAS 9.4.

## Table S3 Summary of baseline covariate degree of missingness and distributions before and after multiple imputation (MI) for the Flatiron Health (FH) newly diagnosed multiple myeloma cohort (n = 1957).

| **Characteristic** | **Flatiron Health Patient Cohort** | | | |
| --- | --- | --- | --- | --- |
|  | **Before multiple imputation** | | **After multiple imputation** | |
|  | **Statistic**† | **Missingness**  **N (%)** | **Statistic*** | **Missingness**  **N (%)** |
| **Age in years, mean (SD)** | 75.2 (5.4) | 0 (0%) | 75.2 (5.4) | 0 (0%) |
| **Sex, N (%)** |  | 0 (0%) |  | 0 (0%) |
| Female | 940 (48.0%) |  | 940 (48.0%) |  |
| Male | 1017 (52.0%) |  | 1017 (52.0%) |  |
| **Race, N (%)** |  | 159 (8.1%) |  | 0 (0%) |
| Black or African American | 297 (16.5%) |  | 326 (16.7%) |  |
| Other | 1501 (83.5%) |  | 1631 (83.3%) |  |
| **First-line therapy (LOT1) initiation year, N (%)** |  | 0 (0%) |  | 0 (0%) |
| 2011 | 99 (5.1%) |  | 99 (5.1%) |  |
| 2012 | 161 (8.2%) |  | 161 (8.2%) |  |
| 2013 | 195 (10.0%) |  | 195 (10.0%) |  |
| 2014 | 211 (10.8%) |  | 211 (10.8%) |  |
| 2015 | 281 (14.4%) |  | 281 (14.4%) |  |
| 2016 | 329 (16.8%) |  | 329 (16.8%) |  |
| 2017 | 300 (15.3%) |  | 300 (15.3%) |  |
| 2018 | 303 (15.5%) |  | 303 (15.5%) |  |
| 2019 | 77 (3.9%) |  | 77 (3.9%) |  |
| **International Staging System (ISS), N (%)** |  | 924 (47.2%) |  | 0 (0%) |
| Stage I | 314 (30.4%) |  | 582 (29.7%) |  |
| Stage II | 377 (36.5%) |  | 700 (35.8%) |  |
| Stage III | 342 (33.1%) |  | 674 (34.5%) |  |
| **Cytogenetic risk, %** |  | 579 (29.6%) |  | 0 (0%) |
| High | 226 (16.4%) |  | 317 (16.2%) |  |
| Standard | 1152 (83.6%) |  | 1640 (83.8%) |  |
| **Eastern Cooperative Oncology Group (ECOG) performance status score, %** |  | 773 (39.5%) |  | 0 (0%) |
| 0 | 364 (30.7%) |  | 605 (30.9%) |  |
| 1 | 509 (43.0%) |  | 841 (43.0%) |  |
| 2 | 235 (19.9%) |  | 389 (19.9%) |  |
| 3+ | 76 (6.4%) |  | 123 (6.3%) |  |
| **Months from multiple myeloma diagnosis to LOT1 start, mean (SD)** | 1.6 (3.0) | 0 (0%) | 1.6 (3.0) | 0 (0%) |
| **Laboratory results** |  |  |  |  |
| Creatinine clearance, mL/min, mean (SD) | 58.6 (26.7) | 264 (13.5%) | 58.7 (26.6) | 0 (0%) |
| Hemoglobin g/dL, mean (SD) | 10.6 (1.9) | 255 (13.0%) | 10.6 (1.9) | 0 (0%) |
| Platelet count, 10^9^/L, mean (SD) | 218.5 (85.4) | 519 (26.5%) | 218.5 (85.8) | 0 (0%) |
| Neutrophil count, 10^9^/L, mean (SD) | 3.9 (3.0) | 1068 (54.6%) | 3.9 (3.2) | 0 (0%) |
| Corrected calcium, mg/dL, mean (SD) | 9.6 (0.9) | 379 (19.4%) | 9.6 (0.9) | 0 (0%) |
| Albumin, g/L mean (SD) | 36.3 (6.8) | 479 (24.5%) | 36.3 (6.7) | 0 (0%) |
| Bilirubin (total), mg/dL, mean (SD) | 0.5 (0.4) | 441 (22.5%) | 0.5 (0.4) | 0 (0%) |
| Serum aspartate aminotransferase, U/L, mean (SD) | 23.5 (14.4) | 393 (20.1%) | 23.4 (14.0) | 0 (0%) |
| Serum alanine aminotransferase, U/L, mean (SD) | 22.0 (19.4) | 415 (21.5%) | 21.8 (18.3) | 0 (0%) |
| **Body mass index, kg/m^2^, mean (SD)** | 27.8 (5.9) | 157 (8.0%) | 27.8 (5.8) | 0 (0%) |

* The summary statistics reflecting the distribution of a covariate prior to imputation are based on the subsample of patients with non-missing values for that covariate. Percentages may not sum to 100% due to rounding. The statistics reflecting covariate distributions after imputation are averaged across 10 imputed datasets; for that reason, patient counts after imputation may not sum exactly to 1957.

ECOG PS, Eastern Cooperative Oncology Group performance status; ISS, International Staging System; LOT1, first-line therapy; MI, multiple imputation; SD, standard deviation. High risk cytogenetics defined as presence of del17p, t(4;14), or t(14;16) detected based on fluorescence in situ hybridization (FISH); standard risk defined as absence of del17p, t(4;14), or t(14;16) based on FISH.

# Results: Details on censoring and loss to follow-up

The average duration of follow-up for the primary on-treatment analysis of PFS was 24.9 months in MAIA and 10.9 months in FH. The table below shows the reasons for the end of follow-up in MAIA and FH.

## Supplemental Table 4 Reasons for end of follow-up in MAIA and Flatiron Health patient cohorts.

| **Reason for end of follow-up** | **MAIA** | **Flatiron Health** |
| --- | --- | --- |
| PFS event (disease progression or death) | 33.4% | 24.7% |
| Lost to follow-up / withdrew consent / last lab assessment for progression (patient censored) | 1.3% | 6.4% |
| Dataset cut-off date or maximum MAIA follow-up  (4 years) reached (patient censored) | 47.6% | 15.2% |
| Initiation of second-line therapy (patient censored) | 2.9% | 10.5% |
| Discontinuation of first-line therapy (patient censored) | 14.8% | 43.3% |

Supplemental Figure 1 below shows the number of patients remaining in the risk set over the follow-up period in MAIA and Flatiron Health.

## Figure S1 Selection of eligible MAIA trial participants and Flatiron Health patients with transplant-ineligible newly diagnosed multiple myeloma.


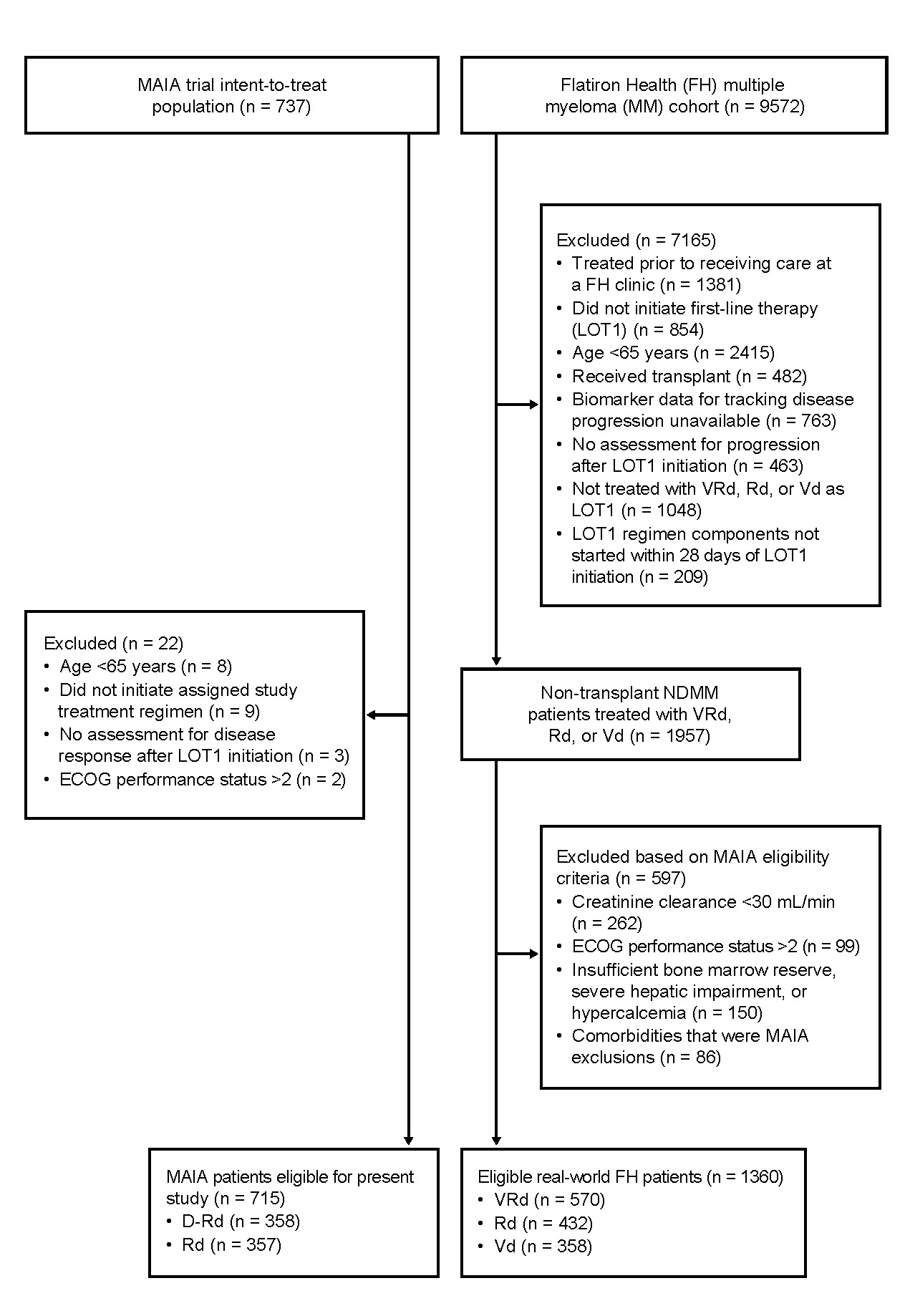


D-Rd, daratumumab-lenalidomide-dexamethasone; ECOG, Eastern Cooperative Oncology Group; FH, Flatiron Health; LOT1, first-line therapy; MM, multiple myeloma; NDMM, newly diagnosed multiple myeloma; Rd, lenalidomide-dexamethasone; Vd, bortezomib-dexamethasone; VRd, bortezomib-lenalidomide-dexamethasone.

## Figure S2 Progression-free survival and loss to follow-up in the primary on-treatment analysis of PFS in the MAIA trial and Flatiron Health patient cohorts by first-line treatment regimen.

Number at risk:

358 299 265 220 45 0

357 257 192 138 19 0

| **MAIA trial** | **Flatiron Health cohort** |
| --- | --- |
| 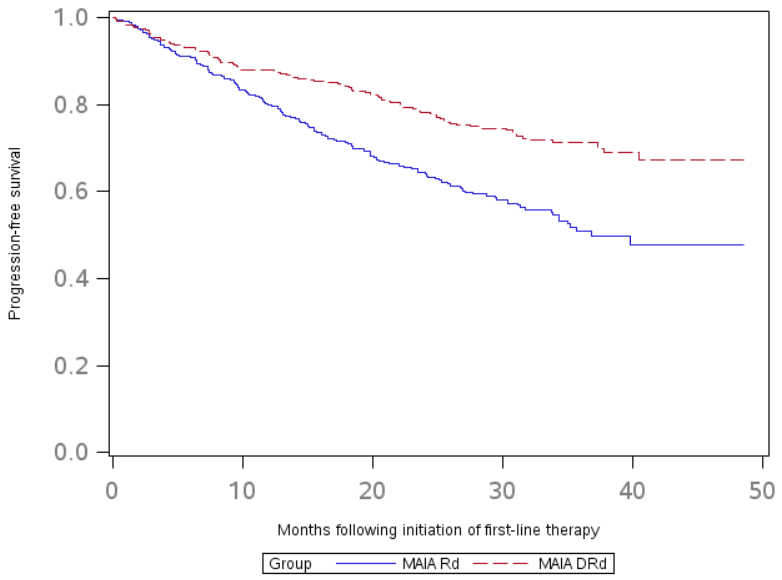 | 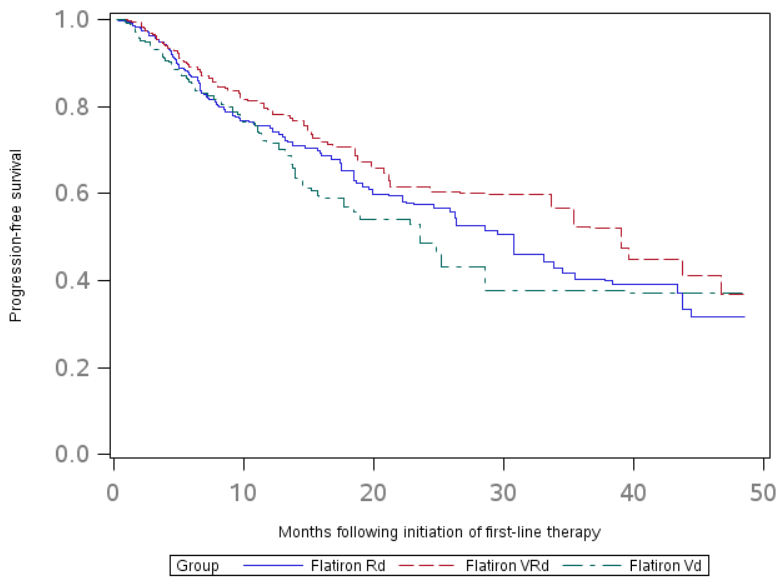  Number at risk:  570 176 69 32 15 0  432 184 87 52 25 0  358 105 21 9 5 0 |

CI, confidence interval; D-Rd, daratumumab-lenalidomide-dexamethasone; HR, hazard ratio; Rd, lenalidomide-dexamethasone; Vd, bortezomib-dexamethasone; VRd, bortezomib-lenalidomide-dexamethasone. Figure reflects results for primary on-treatment analysis of progression-free survival (PFS) with weighting of FH treatment groups to resemble MAIA trial population.

## Figure S3 Overall survival hazard ratios for D-Rd relative to alternative standard-of-care regimens based on direct and indirect treatment comparisons.


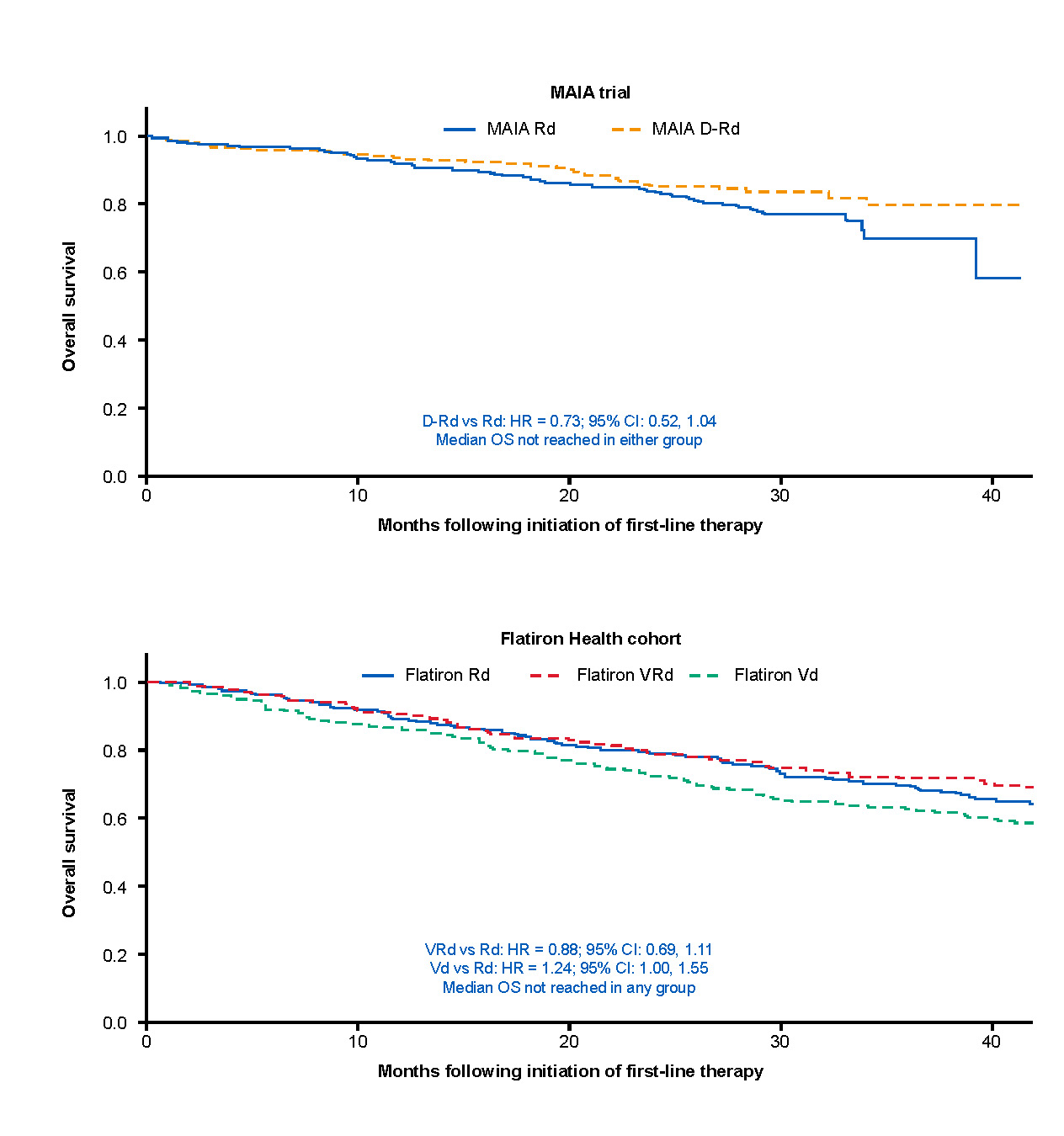


CI, confidence interval, D-Rd, daratumumab-lenalidomide-dexamethasone; HR, hazard ratio; ITC, indirect treatment comparison; Rd, lenalidomide-dexamethasone; Vd, bortezomib-dexamethasone; VRd, bortezomib-lenalidomide-dexamethasone. Figure reflects results for exploratory analysis of overall survival (OS) with weighting of Flatiron Health treatment groups to resemble MAIA trial population.

# References

1. Durie BG, Harousseau JL, Miguel JS, et al. International uniform response criteria for multiple myeloma. Leukemia 2006;20:1467-1473.

2. Rajkumar SV, Harousseau JL, Durie B, et al. Consensus recommendations for the uniform reporting of clinical trials: report of the International Myeloma Workshop Consensus Panel 1. Blood 2011;117:4691-4695.

3. Azur MJ, Stuart EA, Frangakis C, et al. Multiple imputation by chained equations: what is it and how does it work? Int J Methods Psychiatr Res 2011;20:40-49.

4. Facon T, Kumar S, Plesner T, et al. Daratumumab plus lenalidomide and dexamethasone for untreated myeloma. N Engl J Med 2019;380:2104-2115.

5. Bahlis N. Placeholder for 9-month MAIA update presented at ASH 2019. 2019.

6. Richardson PG, San Miguel JF, Moreau P, et al. Interpreting clinical trial data in multiple myeloma: translating findings to the real-world setting. Blood Cancer J 2018;8:109.

7. Phillippo DM, Ades AE, Dias S, et al. NICE DSU Technical Support Document 18: Methods for Population-Adjusted Indirect Comparisons in Submissions to NICE. In: National Institute for Health and Care Excellent (NICE) Decision Support Unit, editor. Sheffield, UK: Decision Support Unit, University of Sheffield; 2016.

8. Signorovitch JE, Sikirica V, Erder MH, et al. Matching-adjusted indirect comparisons: a new tool for timely comparative effectiveness research. Value Health 2012;15:940-947.

9. Brookhart MA, Wyss R, Layton JB, et al. Propensity score methods for confounding control in nonexperimental research. Circ Cardiovasc Qual Outcomes 2013;6:604-611.

10. Austin PC. An introduction to propensity score methods for reducing the effects of confounding in observational studies. Multivariate Behav Res 2011;46:399-424.

11. Funk MJ, Westreich D, Wiesen C, et al. Doubly robust estimation of causal effects. Am J Epidemiol 2011;173:761-767.

12. Mercer AW, Kreuter F, Keeter S, et al. Theory and practice in nonprobability surveys: parallels between causal inference and survey inference. Public Opin Q 2017;81:250-271.

13. Horton NJ, Kleinman KP. Much ado about nothing: A comparison of missing data methods and software to fit incomplete data regression models. Am Stat 2007;61:79-90.
